# Obesity Risk Gene *TMEM18* Encodes a Sequence-Specific DNA-Binding Protein

**DOI:** 10.1371/journal.pone.0025317

**Published:** 2011-09-28

**Authors:** Jaana M. Jurvansuu, Adrian Goldman

**Affiliations:** Structural Biology and Biophysics, Institute of Biotechnology, University of Helsinki, Helsinki, Finland; Florida State University, United States of America

## Abstract

Transmembrane protein 18 (TMEM18) has previously been connected to cell migration and obesity. However, the molecular function of the protein has not yet been described. Here we show that TMEM18 localises to the nuclear membrane and binds to DNA in a sequence-specific manner. The protein binds DNA with its positively charged C-terminus that contains also a nuclear localisation signal. Increase in the amount of TMEM18 in cells suppresses expression from a reporter vector with the TMEM18 target sequence. TMEM18 is a small protein of 140 residues and is predicted to be mostly alpha-helical with three transmembrane parts. As a consequence the DNA binding by TMEM18 would bring the chromatin very near to nuclear membrane. We speculate that this closed perinuclear localisation of TMEM18-bound DNA might repress transcription from it.

## Introduction

According to the NCBI's Entrez Gene search, humans have over 200 proteins named merely transmembrane protein (TMEM). The first publication mentioning TMEM18 appeared in 2008 when *TMEM18* was identified as a terminal oligo-pyrimidine track gene [1]. Terminal oligo-pyrimidine track is an mRNA *cis*-regulatory sequence that inhibits translation from the mRNA, for example, in growth arrested cells. The same year TMEM18 emerged from a screen designed to identify proteins, which enhance neural stem cell migration towards gliomas [2]. In the article it was shown that TMEM18 regulates neuronal stem cell mobility *in vivo* as well as *in vitro*. Abdullah *et al.* found *TMEM18* mRNA to be among the transcripts that correlated with tumorigenicity of human tumour-derived cell lines [3].

From the beginning of 2009 there has been a spate of articles linking *TMEM18* to obesity [4]–[12]. The first two articles were meta-analyses of genome wide association studies of single-nucleotide polymorphism (SNP) and body mass index (BMI) [5], [6]. In these studies tens of thousands of individuals were analysed, which gave exceptional power to detect genes with small effects on BMI. The strongest impact on BMI was with Fat mass and obesity associated (FTO), a gene already identified as an obesity risk [13]. The second best association to BMI was with *TMEM18*. The SNP variant linked to *TMEM18* added 0.26 kg/m^2^ to BMI, which for 170 cm tall adult corresponds to 750 g [6]. For FTO the change in BMI per allele was 0.33 kg/m^2^. Subsequent articles confirmed the role of *TMEM18* as an obesity risk in adult Europeans, extended the linkage to childhood and adolescent obesity, and the obesity of Japanese [8]–[10], [14] [15]. In the articles it was speculated that the detected *TMEM18* expression in brain and particularly in hypothalamus would translate into feeding behaviour.

TMEM18 is a small protein of 140 amino acids. It has a functional nuclear localisation signal and it is predicted to have three transmembrane helices [2]. Although, the studies related to obesity highlight the expression of *TMEM18* in brain, it is expressed robustly in most of the tissues studied in human, mouse, rat, and fruit fly [6], [16], [17] [18]. This abundant expression pattern would imply that the protein has some general function in cells. Moreover, TMEM18 is well conserved during evolution from plants to animals. Yeast and roundworm *C. elegans* are two known exceptions of the sequenced eukaryotes to lack an obvious homologue to *TMEM18* [16]. Thus, TMEM18 seems to be clearly beneficial yet not indispensable.

What does TMEM18 do in the cell? Here we provide evidence and hypothesise that TMEM18 might be involved in gene silencing.

## Results

### Recombinant TMEM18 localises to nuclear membrane

**Figure 1:**
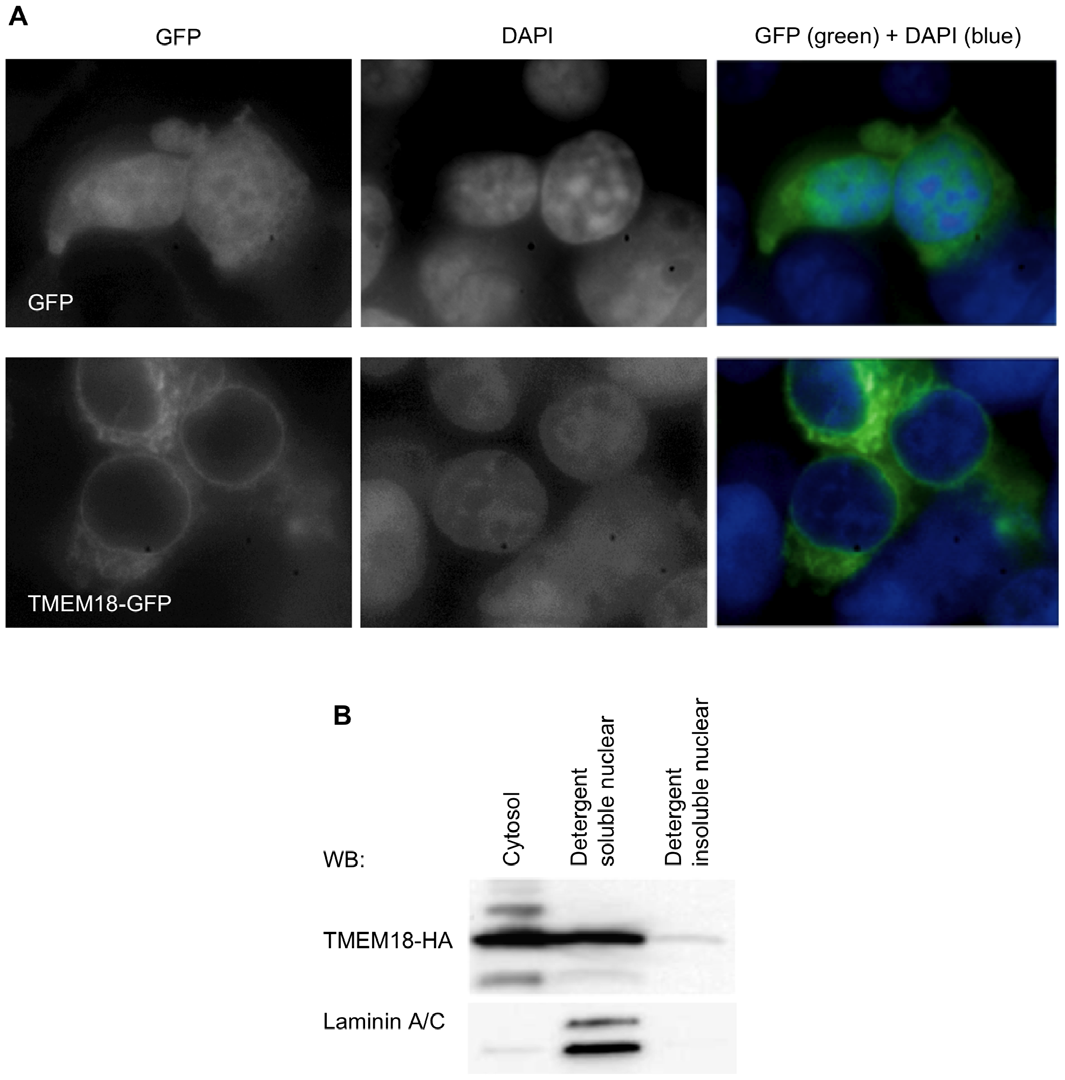
TMEM18 localises to nuclear membrane and cytoplasm. (A) The localisation of TMEM18-GFP in cells was studied by fluorescence microscopy. The cells in the first row express GFP alone and in the second TMEM18-GFP. The first column shows GFP expression, the second DAPI stained nuclei, and the third the superimposition of the two pictures. (B) Western blot analysis of cell fractions from TMEM18-HA overexpressing cells. TMEM18 is mainly in cytosolic and nuclear detergent soluble fractions. A small amount of TMEM18 can be seen also in detergent insoluble nuclear fraction containing proteins bound to DNA. Laminin A/C was used as the control for the purity of the cell fractionation.

We used fluorescence microscopy and cell fractionation to determine the localisation of recombinant TMEM18. Cells were transfected either with a vector expressing GFP or TMEM18-GFP. Nuclei were stained with DAPI before detection by fluorescence microscopy. GFP alone localises throughout the cell, whereas TMEM18-GFP showed a clear ring-like structure around the nucleus (Figure 1A). This suggests that the TMEM18-GFP is in the nuclear envelope. TMEM18-GFP localised also to cytoplasmic structures, most probably to the endoplasmic reticulum (ER).

Next we did a cell fractionation to verify the nuclear localisation of TMEM18. HA-tagged TMEM18 was transfected into cells, and the next day cytosol and nuclei were separated by hypotonic treatment and centrifugation. Nuclear proteins were further separated into detergent soluble and insoluble fractions. The detergent insoluble pellet was treated with acid to release DNA-bound proteins. Laminin A/C is a soluble nuclear protein and was used as a positive control for the purity of nuclear preparation. According to the Western blot (Figure 1B) most of the TMEM18-HA was in the cytosol and detergent soluble nuclear fractions. The cytosolic fraction contains soluble proteins as well as proteins bound to endoplasmic reticulum (ER). The nuclear detergent soluble fraction contains soluble nuclear proteins and proteins bound to nuclear envelope. A small amount of TMEM18 remained in the insoluble nuclear pellet. Detergent insoluble nuclear proteins pellet together with the chromatin.

Recombinant TMEM18 localised to cytoplasm and nuclear membrane. This pattern of localisation resembles that reported for the natural protein [2]. Moreover, we saw that some TMEM18 was also found in nuclear detergent insoluble fraction, which indicates that TMEM18 might be bound to chromatin.

### TMEM18 binds DNA with its C-terminus

**Figure 2:**
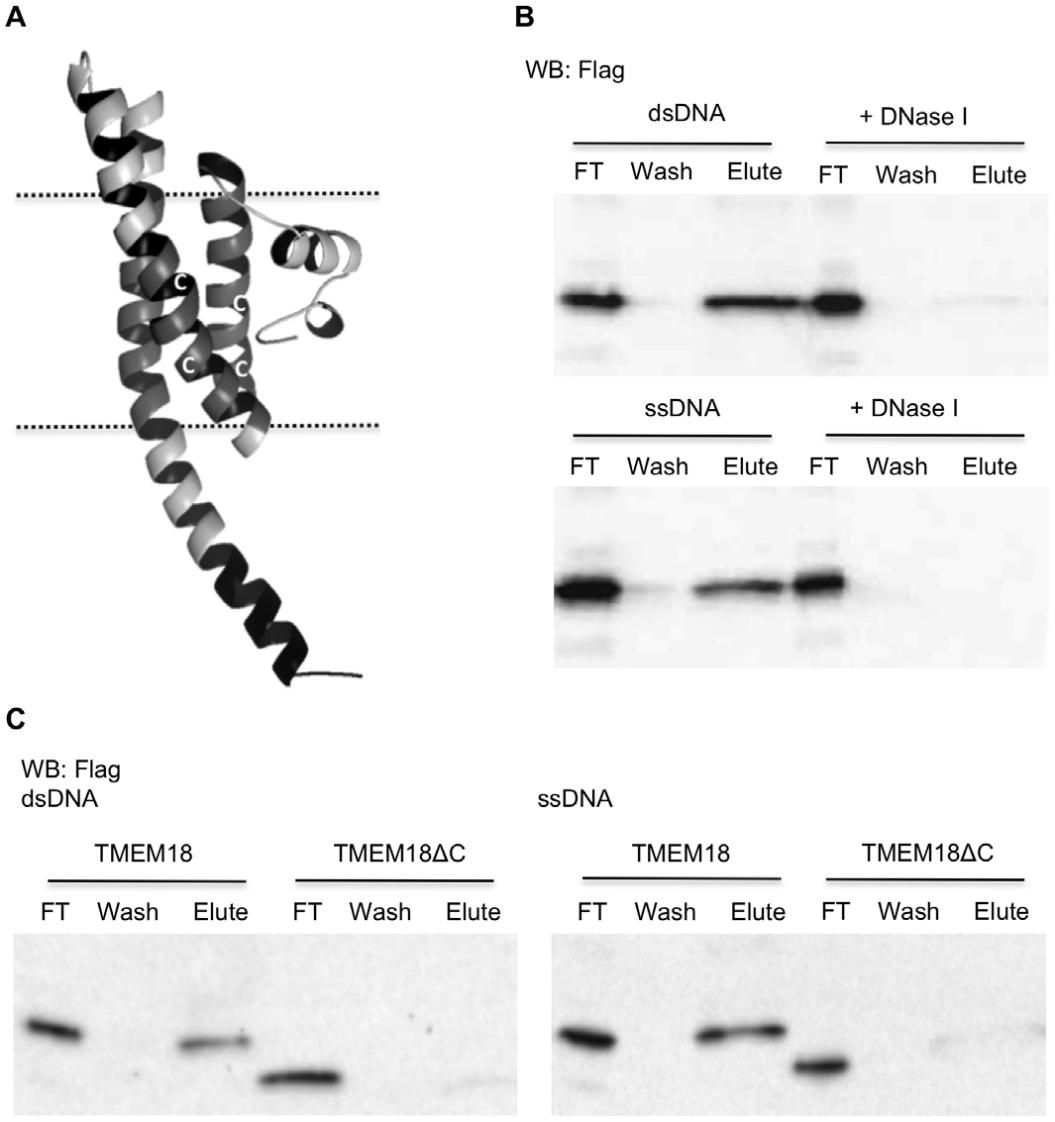
The positively charged C-terminus of TMEM18 binds DNA. (A) Robetta server-predicted (robetta.bakerlab.org) structure model of TMEM18. Dashed lines depict nuclear membrane, the three transmembrane domains are in gray, the C-terminal DNA-binding domain, ERRKEKKRRRKED, is coloured black, and white C's indicate the sites of cysteines. The structural model was edited with PyMOL. (B) TMEM18 binds to dsDNA and ssDNA-cellulose resin. Western blot of Flag-tagged TMEM18 shows the amount of TMEM18 in flow through (FT), wash, and elute. DNase I treatment of the DNA-cellulose resins erased the TMEM18 binding demonstrating that TMEM18 does not bind to the cellulose matrix. (C) TMEM18 lacking the last 13 C-terminal amino acids was unable to bind DNA-cellulose. Western blot results are shown for both dsDNA and ssDNA-cellulose binding assays.

Robetta server uses ROSETTA software to model three-dimensional protein domains either by fragment homology modelling or by an *ab initio* protocol for proteins, like TMEM18, without Protein Data Base homologues [19]. The Robetta predicted TMEM18 to consist mainly of alpha helixes (Figure 2A). The C-terminal part of TMEM18 has a nuclear localisation signal and thus should be inside the nucleus. The C-terminus has an array of large hydrophilic amino acids (ERRKEKKRRRKED) and must thereby extend outwards from the membrane (Figure 2A). This protruding part with several positive amino acids, i.e. lysines and arginines, is a good candidate to bind DNA. Because the ROSETTA software does not take into account the lipid membrane, the mainly non-structured N-terminus shown to be inside the membrane in Figure 2A is unlikely to be in that position.

We used chromosomal DNA linked to cellulose to study the binding of TMEM18 to DNA. All DNA binding experiments included the non-ionic detergent, dodecyl maltoside, in the buffers to ensure native conformation of TMEM18. Protein extracts from TMEM18-HA overexpressing cells were incubated with DNA-cellulose, washed, and protein bound to the DNA was eluted with high salt concentration. As shown in Figure 2B, TMEM18 could be eluted from both single and double-stranded DNA-cellulose. Dnase I treated DNA-cellulose was used as a negative control to demonstrate that the binding was to DNA and not to the cellulose matrix. As expected, when the DNA was removed from the cellulose resin TMEM18 no longer bound (Figure 2B).

Next we wanted to test whether the positively charged C-terminus is the DNA-binding domain. We constructed C-terminal deleted TMEM18, which lacks the last 13 C-terminal amino acids and studied its binding to the DNA-cellulose. TMEM18ΔC bound neither single nor double-stranded DNA (Figure 2C). The C-terminus is indispensable for TMEM18 DNA binding.

### TMEM18 oligomerises

**Figure 3:**
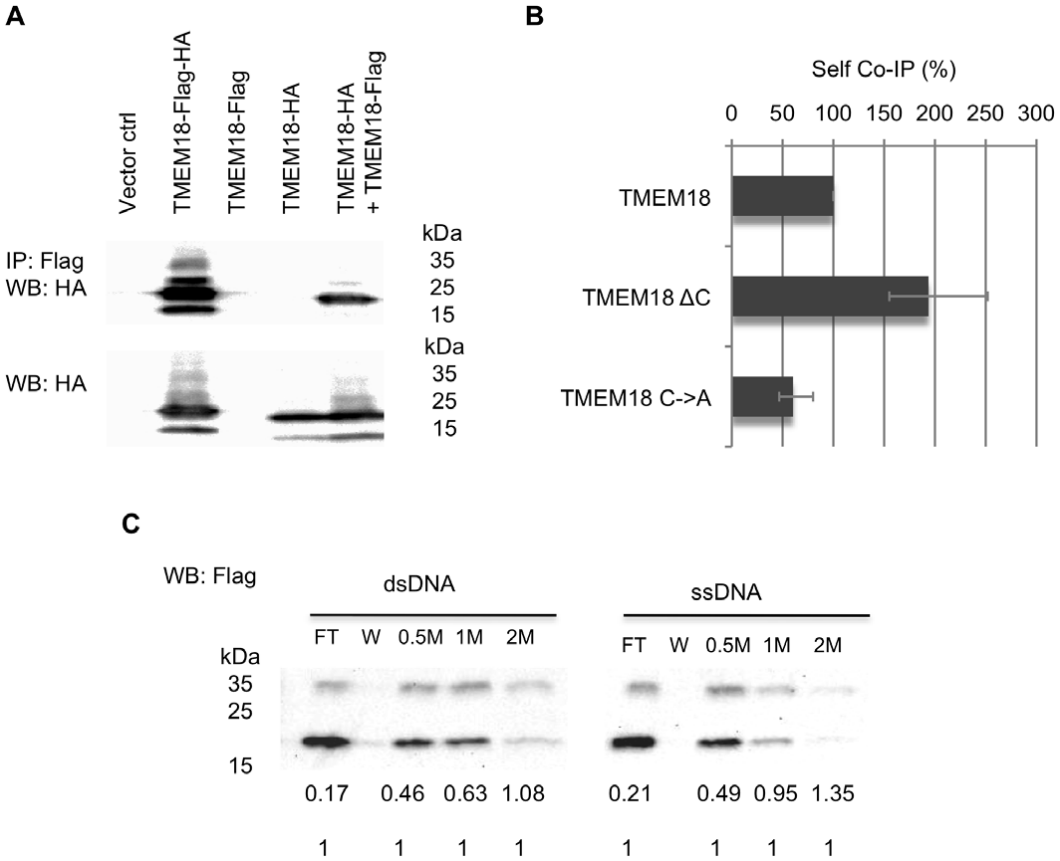
TMEM18 oligomerises independently of the DNA-binding domain. (A) Flag-tagged TMEM18 binds HA-tagged TMEM18 as shown in a Western blot. Untransfected cells (empty ctrl), vector transfected (Vector ctrl), or HA and Flag double-tagged TMEM18 (TMEM18-Flag-HA), Flag-tagged TMEM18 (TMEM18-Flag), HA-tagged TMEM18 (TMEM18-HA), or both HA and Flag-tagged TMEM18 expressing cells were immunoprecipitated with Flag and detected with anti-HA antibody. The relative amounts of HA-tagged TMEM18 in the cell extracts before immunoprecipitation are shown under the immunoprecipitated samples. (B) TMEM18 C-terminus is not needed for self-binding but the interaction is facilitated by cysteines. Co-immunoprecipitation assays were done using TMEM18-Flag with TMEM18-HA, C-terminal deletion TMEM18 (TMEM18ΔC), or TMEM18 with all the four cysteines mutated into alanines (TMEM18C→A). The samples were immunoprecipitated with Flag antibody, Western blot was probed with HA antibody, and the signal intensities were estimated from the blot. The results are a combination of three separate experiments and the error bars show the experimental variation. (C) TMEM18 dimerisation facilitates DNA binding. TMEM18 was purified from insect cells and bound to DNA-cellulose. Unbound proteins were in flow through (FT). Samples on DNA-cellulose were washed (W) and then eluted with increasing salt concentration (0.5 M, 1 M, and 2 M). The numbers under the Western blot indicate the ratio of the dimer form (approx. 34 kDa) of TMEM18 to the monomer form (approx. 17 kDa).

Dimerisation is a common phenomenon for DNA-binding proteins. To study if TMEM18 binds itself we assayed whether Flag-tagged TMEM18 can immunoprecipitate HA-tagged TMEM18. To control for nonspecific binding, cells were transfected with empty vector, TMEM18-HA, or TMEM18-Flag. Double-tagged TMEM18-HA-Flag was a positive control for the experiment. Protein extracts were immunoprecipitated with anti-Flag beads and probed with anti-HA antibody. TMEM18 binds itself as shown by positive signal for HA in co-transfection sample of TMEM18-HA and TMEM18-Flag (Figure 3A). None of the negative controls, empty vector or single tagged TMEM18 alone, showed the correct size signal in the Western blot. The relative amounts of TMEM18-HA in cell extracts before immunoprecipitation are shown in Figure 3A.

TMEM18 C-terminus has been predicted to have a coiled-coil oligomerisation domain [16]. We did the same self-co-immunoprecipitation experiment to the C-terminal deleted TMEM18. Surprisingly, lack of the C-terminus seemed to facilitate the TMEM18 self-association (Figure 3B). The predicted coiled-coil is very short (three heptad repeats) and possibly an artefact of the coiled-coil prediction software due to the high charge content of the sequence. At least, the presumed coiled-coil has no role in TMEM18 oligomerisation.

TMEM18 has altogether four cysteines, two of which are in the first transmembrane domain and the other two in the second transmembrane domain (Figure 2A). Mutating all the cysteines into alanines reduced the TMEM18 oligomerisation, but did not completely erase it. This result suggests that disulphide bonding is involved in the self-binding but is not the only factor. TMEM18 self-association could thus occur through the transmembrane parts inside the lipid membrane. This would explain why dimer and trimer sized TMEM18 can sometimes be seen in SDS protein gels when the protein is heavily overexpressed. Mild denaturing conditions are used for membrane proteins in gels to ensure that they do not aggregate through their hydrophobic parts in a loading buffer, therefore it is possible that TMEM18 remains linked via its membrane domains during SDS-PAGE.

Although the DNA-binding ability and oligomerisation are not physically related, dimerisation seemed to stabilise TMEM18 onto DNA (Figure 3C). TMEM18 was produced in insect cells and incubated with the DNA-cellulose resin, washed, and eluted with salt gradient. In the Western blot the proportion of dimer-sized TMEM18 increased the more tightly TMEM18 was bound to DNA, i.e., how high a salt concentration was needed to elute the protein. The ratio of monomeric to dimeric TMEM18 in the flow through was almost 10 to 1, whereas for the protein still bound to dsDNA after 1 M salt wash it was equal. The trend was similar for ssDNA binding.

### TMEM18 prefers binding to GCT trimers

**Figure 4:**
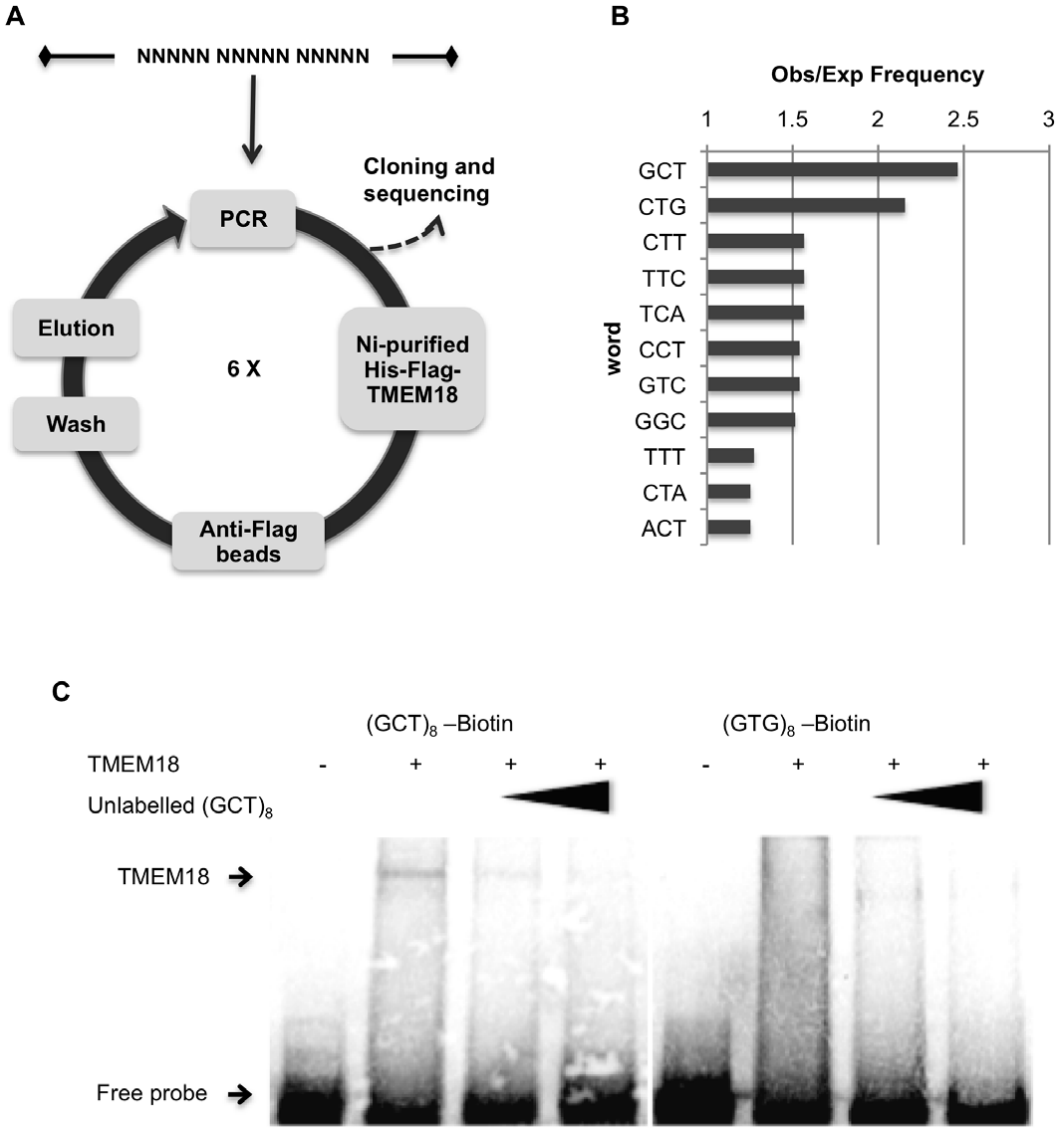
TMEM18 binds to GCT sequence. (A) SELEX was used to identify the specific sequences TMEM18 binds. The TMEM18 binding oligonucleotides were enriched by six rounds of SELEX before they were cloned and sequenced. (B) The obtained sequences were analysed by comparing the observed frequency of three nucleotide words to that expected of a random distribution (Wordcount at Mobyle@pasteur). The results show that 11 nucleotide triplets were found to have higher than expected frequency and of these GCT was the most enriched by ratio of almost 2.5. (C) Biotin-labelled oligonucleotides with eight repeats of either GCT or non-target sequence GTG were used in EMSA to confirm the SELEX results. In competition experiment an unlabelled (GCT)_8_ oligonucleotide was used in increasing amounts (2.5 and 6.25-fold excess) shown by a black triangle. An arrow indicates the unbound free probe as well as the TMEM18 shifted oligonucleotides.

Although TMEM18 binds both ssDNA and dsDNA, we decided to concentrate on its binding to dsDNA because it is a more plausible target for a nuclear protein. We used systematic evolution of ligands by exponential enrichment (SELEX) to identify the sequences that TMEM18 favours. Double-stranded oligonucleotide with 15 random nucleotides was incubated with nickel column-purified histidine and Flag-tagged TMEM18. The protein-DNA complexes were bound with anti-Flag beads. The beads were washed and the DNA bound to TMEM18 was eluted and amplified by PCR. This selection procedure was repeated six times before cloning the oligonucleotides into a vector for sequencing (Figure 4A). Sequencing results from 23 different oligonucleotides were analysed by Wordcount (Mobyle@pasteur) [20]. Because the TMEM18 DNA-binding domain is one alpha helix and most of the protein in these conditions would bind as a monomer, we expected the target sequence to be 3–4 nucleotides. Wordcount was set to identify three letter words, which observed frequency was higher than would be expected in a random DNA sequence (Figure 4B). Only two sequences were twice as frequent as expected, GCT and CTG. No clear sequence preference was found by analysing four-letter words. It could be possible that the sample size was too small to identify longer than three nucleotide targets.

The SELEX assay detected GCT as the most frequent nucleotide trimer in the TMEM18-bound oligonucleotides. Electro-phoretic mobility shift assay (EMSA) was used to prove that TMEM18 has a specific affinity to GCT. Double stranded oligonucleotide with eight repeats of GCT linked to biotin was used as a probe with purified TMEM18 protein. TMEM18 bound (GCT)_8_ oligonucleotide and the shift was erased with unlabeled competing (GCT)_8_ oligonucleotide (Fig. 4C). When the concentration of unlabeled competing oligonucleotide was 6 times in excess the shift was not detectable. As a negative control we used nucleotide trimer GTG, which was seen in the SELEX assay three times more rarely than expected. Accordingly, TMEM18 did not bind biotin-(GTG)_8_ oligonucleotide in EMSA.

### TMEM18 suppresses transcription

What consequence does TMEM18 DNA binding have in cells? Luciferase vector without any enhancer elements and the same vector with 15 repeats of GCT upstream of the SV40 promoter were used to study the effects of TMEM18 on transcription. We transfected the cells first with an empty control vector or a vector expressing TMEM18 or TMEM18-C-JUN and eight hours later the luciferase vectors. In the TMEM18-C-JUN construct, the TMEM18 DNA-binding domain was replaced by that of c-JUN (RKRMRNRIAASKSRK RK). C-JUN and TMEM18 DNA-binding domains resemble each other in size and that they both contain an embedded nuclear localisation signal. One major difference between c-JUN and TMEM18, which might affect their DNA binding, is that c-JUN is a soluble protein. C-JUN binds DNA at the sequence TGAC [21]; this sequence of nucleotides was not among TMEM18 binding targets.

**Figure 5:**
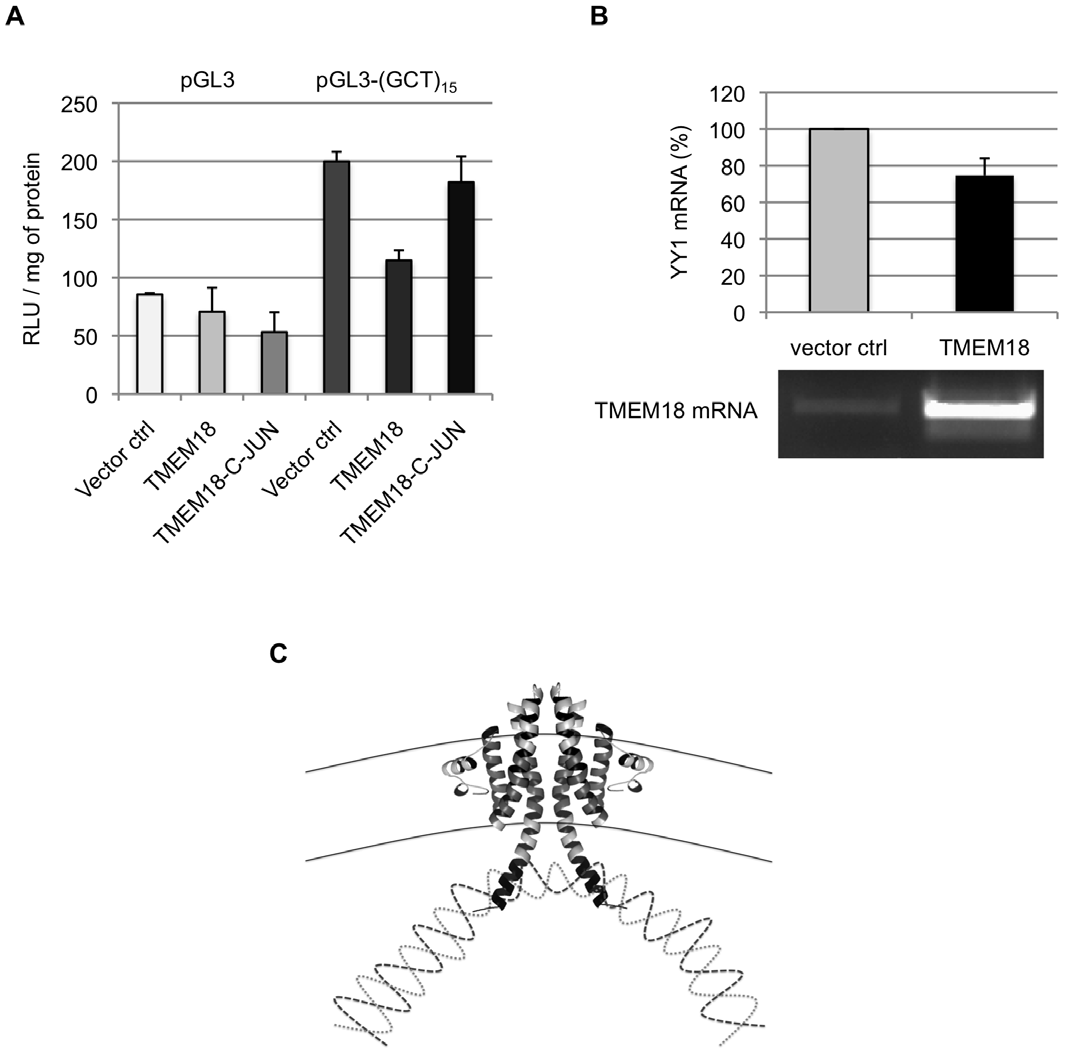
TMEM18 suppresses gene expression. (A) An enhancerless reporter vector pGL3 (light gray bars) and the same vector with 15 repeats of GCT upstream of the promoter (dark gray bars) were used in the luciferase assay. Luciferase readings (RLU / mg of protein) were recorded one day after transfection of the reporter vector to empty vector (Vector ctrl), TMEM18, or TMEM18-C-JUN expressing cells. In TMEM18-C-JUN the c-JUN DNA-binding domain, RKRMRNRIAASKSRKRK, replaced the last 13 C-terminal amino acids of TMEM18. The experiment was done in triplicate and the error bars indicate the standard deviation. (B) TMEM18 inhibits YY1 expression. Cells were transfected with an empty or TMEM18 expressing vector and analysed two days later. TMEM18 and YY1 mRNA levels were normalised to UBC. The quantative real-time PCR results for YY1 are shown as a bar chart and the TMEM18 mRNA levels are visualised in an ethidium bromide stained agarose gel. The results are from three separate experiments and the error bars denote standard deviation. (C) Hypothetical model of how TMEM18 suppresses gene transcription by bringing the chromatin to the nuclear periphery. Two TMEM18 molecules are shown at the nuclear membrane indicated by black solid lines, interweaving dashed lines represent DNA.

GCT repeats seem to function as an enhancer element as luciferase expression more than doubled following addition of (GCT)_15_ to the reporter vector (Figure 5A). When TMEM18 was overexpressed in cells luciferase expression levels were reduced significantly from the pGL3-(GCT)_15_ but not from the unmodified control vector. TMEM18 with C-JUN nuclear localisation signal and DNA-interaction domain was not able reduce the luciferase transcription suggesting that the suppression is due to TMEM18 DNA-binding domain interaction with its target sequence in the luciferase reporter vector.

Jurvansuu et al. reported that the when TMEM18 overexpression increased cell migration, it correlated with augmented transcription of C-X-C chemokine receptor type 4 (CXCR4) [2]. CXCR4 transcription is repressed by transcription factor Yin Yang 1 (YY1) [22]. We hypothesised that TMEM18 overexpression leads to increased transcription of CXCR4 through TMEM18 mediated repression of the YY1 gene. Cells were transfected with empty or TMEM18 expressing vector and cytoplasmic RNA was isolated two days later. YY1 and TMEM18 expression was normalised to UBC mRNA levels. Increase in TMEM18 repressed YY1 expression on average by 25% (Figure 5B). According to our cross-linking chromatin immunoprecipitation (ChIP) assay, immunoprecipitation of TMEM18 recovers YY1 promoter sequences (Figure S1). This result suggests that some TMEM18 is on YY1 promoter and could thus affect its function. Although the effects on YY1 expression might still be due to indirect interactions, it does demonstrate that TMEM18 overexpression inhibits transcription from chromatin and produces consistent transcriptional changes in different cell lines.

## Discussion

*TMEM18* has been implicated in complex phenomena like cancer, cell migration, and obesity [2] [3], [5], [6]. Here we have shown that TMEM18 localises to the nuclear membrane and binds DNA with its C-terminus in a sequence-specific manner. The GCT nucleotide triplet that TMEM18 prefers to bind may appear inadequate on a genomic scale to create specific interactions. C-JUN has a preferred sequence of four nucleotides, but when it dimerises with proteins of JUN, FOS, ATF, or MAF protein families to assemble the AP-1 transcription factor, this complex has affinity to response elements ranging from 7 to 14 base pairs [23]. TMEM18 binds itself and forms dimers that seem to bind DNA with a higher affinity than monomers. Similarly to c-JUN, this TMEM18 oligomerisation would increase selectivity by extending the length of its target sequence. Also, potential TMEM18-interacting proteins could further modify the specificity of DNA binding.

In our experiments TMEM18 is capable of repressing transcription through its target sequence in a reporter vector. Many inner nuclear membrane proteins affect gene transcription, although typically this occurs in a chromatin-independent manner: nuclear membrane proteins sequester transcription factors to the nuclear periphery and thus repress or activate transcription [24]. The only known exception to this is nuclear (factor erythroid 2)-related factor 1 (NRF1), which is a nuclear membrane protein activating transcription through antioxidant-responsive elements [25]. Both NRF1 and TMEM18 are predicted to have three transmembrane domains, though the former protein is 85 kDa in size whereas TMEM18 is only 17 kDa. The larger size of NRF1 means that when the protein binds to chromatin, there is still space for interactions with transcription machinery. TMEM18 bound to DNA would leave no space between the DNA and the nuclear membrane, making it impossible to large protein complexes to interject. We speculate that this model could explain why TMEM18 seems to repress transcription (Fig. 5C). The TMEM18 target sequence functions also as a transcriptional enhancer element in cells, which is interesting because it implies that there might be a competition for the target site between TMEM18 and a transcription activator.

Alternatively, the inhibition of transcription could be just a by-product of TMEM18 holding onto DNA. Inactive heterochromatin is usually perinuclear and it has been thought that this could be due to the localisation of transcription repressors there or that the nuclear membrane would somehow work as a scaffold to pack chromatin [26]. This nuclear organisation of chromatin is important as the release of perinuclear chromatin has shown to result in genomic instability [27]. If TMEM18 is a chromatin organisation factor the short target sequence would be adequate, even appropriate, for its function.

In conclusion, TMEM18 is a nuclear membrane protein that binds DNA and suppresses transcription. Genome wide search, like ChIP-on-chip, for the natural DNA targets of TMEM18 would help to define the binding sequence and to clarify whether the protein has an active or passive role in transcriptional regulation. Additionally this kind of experiment could reveal new pathways leading to obesity.

## Materials and Methods

### Cells, transfection, plasmids, baculoviruses, TMEM18 mutations, and Western blotting

293T [28] and U2OS [29] cells were cultured in Dulbecco's modified Eagle's medium supplemented with 5% fetal bovine serum (Lonza), and penicillin-streptomycin (Gibco). Cells were transfected with either TransIT-2020 (Mirus) or Attractene (Qiagen) according to the manufacturer's instructions. Sf9 and Tn5 cells (Invitrogen) were maintained in HyQSFX-medium (HyClone) supplemented with gentamycin.

For all the overexpression experiments in mammalian cells TMEM18 constructs were cloned into pMONO-blasti-mcs vector (InvivoGen). TMEM18-GFP expression vector was created by cloning GFP from pBabe-GFP (Addgene) into pMono-TMEM18 vector. PGL3-promoter vector (Promega) was used in luciferase assays. For insect cell expression, TMEM18 was cloned into pK509.3 vector [30]. Baculoviruses were produced according to the Bac-to-Bac protocol (Invitrogen).

All the oligonucleotides used in this study were purchased from Sigma or IDT. TMEM18 mutations were constructed using site-directed mutagenesis (QuickChange, Stratagene) and had a C-terminal HA or Flag-tag or both. TMEM18ΔC lacks the last 13 amino acids from the C-terminus. TMEM18C→A has all the cysteines (C38, C43, C58, and C65) mutated into alanine. TMEM18-C-JUN has the last 13 C-terminal amino acids replaced by RKRMRNRIAASKSRKRK sequence of C-JUN.

Horseradish peroxidase (HPR)-conjugated secondary antibodies used in this study were from Santa Cruz Biotechnology. Amersham ECL Plus™ Western Blotting Detection Reagent was used in detection of HRP signal (GE healthcare).

### Microscopy

The 293T cells growing on L-poly-lysine (Sigma) coated cover slips were transfected with pBabe-GFP or pMONO-TMEM18-GFP using TransIT-2020. Two days after transfection the cells were washed with PBS, fixed with 4% paraformaldehyde (Sigma), stained with DAPI (Sigma), and mounted into Mowiol-DAPCO (Fluka). Slides were examined with Leica DMIRE2 microscope with 100× magnification. Images were recorded in black and white; the colour was added with ImageJ-program.

### Cell fractionation

Cell fractionation into cytoplasm and nuclei was done according to Lamond lab protocol (www.lamondlab.com/f7nucleolarprotocol.htm). Shortly, 293T cells were plated a day before transfection with TransIT-2020. One-day post transfection cells were washed with PBS, resuspended into hypotonic buffer, Dounce homogenised, and centrifuged. The supernatant was cytosolic fraction and the pellet was impure nuclear fraction. The nuclear fraction was further purified by sucrose gradient and then resuspended into radio-immunoprecipitation assay (RIPA) buffer, after which the sample was centrifuged at 10 000 rpm for 10 minutes at 4°C. The supernantant was detergent soluble fraction. The pellet was treated with 0.2 M HCl to release DNA bound proteins; this was the detergent insoluble fraction. All the fractions were kept of the same volume. The samples were used for Western blotting with anti-Laminin A/C (N-18, Santa Cruz Biotechnology) and anti-HA (H6908, Sigma) antibodies.

### DNA binding

The deoxyribonucleic acid-cellulose resins (Sigma) were stored in 4 ml of 10 mM Tris pH 7.9, 1 mM EDTA per gram of resin. 293T cells were plated on a six-well plate and the TMEM18 constructs were transfected with TransIT-2020 the next day. The proteins were extracted one day post-transfection. In short, cells were washed three times with phosphate buffered saline (PBS) and then suspended into RIPA buffer supplemented with protease inhibitors (cOmplete, Roche) and 0.5 mM DTT. The cell suspension was sonicated, centrifuged, and the supernatant was collected for the DNA binding assay. The protein extracts were incubated in 10 mM Tris pH 7.4, 0.1 M NaCl with 1/100 vol of DNA-cellulose resin rotating at 4°C. The matrix was washed with 10 mM Tris pH 7.4, 0.2 M NaCl and proteins were eluted with 10 mM Tris pH 7.4, 2 M NaCl. The samples were incubated at 65°C in SDS loading buffer before loading them onto SDS-PAGE. TMEM18 was detected by Western blot with Anti-Flag antibody (F3165, Sigma).

Dnase I treatment of the matrix was done as follows: the matrix was washed three times with 10 mM Tris pH 7.5, 0.2 M NaCl and incubated with 10 mg/ml of Dnase I (Sigma) in 10 mM Hepes pH 7.9, 10 mM KCl, 1.5 mM MgCl_2_ for 15 minutes at room temperature (RT). Then the matrix was washed three times with 10 mM Tris pH 7.4, 1 mM EDTA and resuspended into 10 mM Tris pH 7.5, 0.1 M NaCl.

### TMEM18 purification from insect cells

TMEM18 produced in Sf9 or Tn5 insect cells was isolated using hypotonic treatment, cell fractioning, and nickel-column purification. The TMEM18 expressed in insect cells had an N-terminal Flag-tag followed by eight histidine repeats. In short, Sf9 or Tn5 cells were infected with high titer of TMEM18 baculoviruses and the cells were cultured at 27°C for three days, after which the cells were spun down and washed once with Tris-buffered saline (TBS). The cells were then resuspended into 10 mM Tris-HCl pH 7.4, 10 mM NaCl, 1.5 mM MgCl_2_, 0.5 mM DTT and protease inhibitors and incubated at 4°C for 1 hour before the cells were disrupted with a Douncer homogeniser. Nuclei were collected by centrifugation and resuspended into PBS, 1% dodecyl maltoside (DDM, Anatrace). Then the nuclei were sonicated and insoluble material was spun down. Nuclear extracts were incubated in PBS, 40 mM imidazole and Ni-sepharose-beads (GE Healthcare) for 1 hour at 4°C, after which the beads were washed with PBS, 80 mM imidazole, 0.05% DDM. Protein was eluted with 10 mM Hepes, 0.5 M imidazole, 0.05% DDM and used immediately for the DNA binding, SELEX, or EMSA. The insect cell produced TMEM18 was probed in Western blot with anti-Flag antibody from Sigma (F3040).

### Co-immunoprecipitation

Empty vector, TMEM18-HA, TMEM18-Flag, or TMEM18-HA-Flag were transfected into 293T cells and the cell extracts were prepared the next day as described under “DNA binding”. Samples of cell extracts were probed with HA-antibody. The extracts were rotated in RIPA buffer with EZview™ Red ANTI-FLAG® M2 Affinity Gel (Sigma) at 4°C for one hour. Beads were washed with RIPA buffer plus 200 mM NaCl and eluted with SDS loading buffer. Samples were separated on SDS-PAGE, blotted and probed with anti-HA antibody. The protein ratios in Western blot were analysed with Molecular Imager ChemiDoc™ XRS system (BioRad) and QuantityOne (BioRad).

### Systematic Evolution of Ligands by Exponential Enrichment (SELEX)

TMEM18 was purified from Sf9 insect cells as described in section “TMEM18 purification from insect cells”. The SELEX bait oligonucleotide sequence was 5′ CTGCAGTTGCACGATATCNNNNNNNNNNNNNNNGTCGACTGAATTCGCCTC. Nickel column-purified TMEM18 was rotated with 2.5 µg of double stranded bait oligonuclotide in 10 mM Tris pH 7.4, 0.1 M NaCl, 0.05% DDM at 4°C for 1 hour. EZview™ Red ANTI-FLAG® M2 affinity gel beads were added to the sample and the mixture was incubated for one more hour, before washing the beads with 10 mM Tris pH 7.4, 0.2 M NaCl, 0.05% DDM and eluting the bound DNA with 10 mM Tris pH 7.4, 2 M NaCl, 0.05% DDM. DNA was purified with NucleoSpin Extract II kit (Macherey-Nagel) and half of the elution was used for PCR. The PCR oligos were 5′ CTGCAGTTGCACGATATC and 5′ GAGGCGAATTCAGTCGAC. The PCR program was as follows: 93°C for 3 minutes, 93°C for 30 seconds, 55°C for 30 seconds, 72°C for 10 seconds. The PCR cycle was repeated 25 times. Then 2.5 µg of PCR product was used directly to a next round of SELEX. SELEX was repeated six times before the PCR product was digested with SalI and EcoRV, cloned into pMONO vector, and sequenced.

### Electrophoretic mobility shift assay (EMSA)

TMEM18 was purified from Tn5 insect cells as described in section “TMEM18 purification from insect cells”. EMSA oligonucleotides, (GCT)_8_ with and without 5′ biotin, (AGC)_8_, (GTG)_8_ with 5′ biotin, and (CAC)_8_, were purchased from IDT. Oligonucleotides were annealed in equimolar concentration by heating them at 95°C for 5 minutes and then letting them cool down gradually to RT.

TMEM18 and 400 nM of biotinylated oligonucleotides were incubated in EMSA buffer (10 mM Tris pH 7.4, 50 mM KCl, 1 mM DTT, and 0.05% DDM) for 30 minutes at RT. In the competition reactions 1 and 2.5 µM unlabelled (GCT)_8_ oligonucleotides were added to the reactions. The reactions were loaded into pre-run 4–20% Tris-Borate-EDTA (TBE) gel (BioRad) and run in 0.5× TBE buffer (44.5 mM Tris, 44.5 mM Boric acid, 1 mM EDTA). DNA was electrophoretically transferred onto positively charged nylon membrane (Amersham) and crosslinked by UV at 254 nm. Chemiluminescent Nucleic Acid Detection Module (Thermo Scientific) was used to visualise the biotin-labelled oligonucleotides as recommended by the manufacturer.

### Luciferase assay

Repeat sequence of 15 GCTs was inserted into the SmaI site of pGL3-promoter vector. 293T cells were plated on a 24-well plate at 90% confluency and transfected next day first with pMONO or pMONO-TMEM18 and 8 hours later with luciferase reporter vectors. Measurements of the luciferase expression were done 16 hours after the last transfection with 1254 Luminova (Bio-Orbit) using Luciferase Assay System (Promega) as recommended by the manufacturer. Protein concentration was measured by Bradford assay (BioRad).

### Real-time PCR

U2OS cells were transfected with pMONO or pMONO-TMEM18 and cytoplasmic RNA was isolated two days after the transfection with RNeasy kit (Qiagen). 1 µg of RNA was used for reverse transcription (Ominiscript, Qiagen). Real-time PCR was done using QuantiFast SYBR green PCR kit (Qiagen) as recommended by the manufacturer. The following primers were used: for YY1 5′-AGTGGGAGCAGAAGCAGG 5′-TCATGGCCGAGTTATCCC [31], and for UBC 5′-ATTTGGGTCGCGGTTCTTG 5′-TGCCTTGACATTCTCGATGGT [32]. PCR for TMEM18 was done using Pfu polymerase (Fermentas) as recommended by the manufacturer using primers: 5′-ATGCCGTCCGCCTTCTCTGTC and 5′-TCAGTCTTCT TTCCTTCTCC.

## Supporting Information

Figure S1 **TMEM18 binds YY1 promoter sequences.** Chromatin immunoprecipitation was done to empty control and TMEM18-Flag expressing cells. The results are from three separate chromatin immunoprecipitation experiments. A standard curve was used to assign the samples a relative value. The ChIP sample values were divided by corresponding input sample values and these numbers were compared between control and TMEM18 expression samples so that mean control value was set to 100%. Standard error is indicated (N_ctrl_ = 15, N_TMEM18_ = 12). ChIP was done according to the Abcam protocol (www.abcam.com/index.html?pageconfig=resource&rid=11698). In short, U2OS cells were plated on 10 cm plates and transfected (Attractene, Qiagen) with pMONO-TMEM18-FLAG or empty pMONO. Two days after tranfection cells were cross-linked and sonicated three minutes with a microtip at 40% power (Bandelin Sonoplus). An input control sample of 50 µl was removed after sonication. Flag-tagged TMEM18 was immunoprecipitated with EZview™ Red ANTI-FLAG® M2 Affinity Gel (Sigma) at 4°C for two hours. The samples were washed, eluted, and cross-linking was reversed by 5 hour incubation at 65°C. The DNA was purified by DNA purification kit (Macherey-Nagel). Binding of TMEM18 to the YY1 promoter region was assayed by real-time PCR for input and immunoprecipitated samples from TMEM18-Flag expressing and empty control samples. QuantiFast SYBR Green kit with primers 5′ CTGCAATGTAACTCATTCAGGAAG and 5′ GTGCCTGTTTCCCCGTAGAT was used in PCR (Stratagene Mx3005P). Dilutions of input samples were used to construct a standard curve.
(TIF)
